# Reliability, Validity, and Clinical Interrelationships of Lower-Extremity Screening Measures in College Athletes

**DOI:** 10.3390/diagnostics16142256

**Published:** 2026-07-19

**Authors:** Aylin Ardalı, Deren Kalfaoğlu, Melih Köksal, Ezgi Tuna Erdoğan

**Affiliations:** 1 School of Medicine, Koç University, Istanbul 34450, Türkiye; 2 Department of Physiology, School of Medicine, Koç University, Istanbul 34450, Türkiye

**Keywords:** foot posture, dynamic balance, navicular drop, hallux dorsiflexion, body mass index, athlete screening

## Abstract

**Background/Objectives:** Modifiable intrinsic factors such as foot posture, hallux mobility, dynamic balance, and body mass index (BMI) are widely assessed in athletic screening, but their reliability and interrelationships remain unclear. The aim of the study was to evaluate the reliability of foot posture, hallux mobility, dynamic balance, Q-angle, and navicular drop measures in collegiate athletes and to examine the relationships among these measures and BMI. The concurrent, discriminant, and predictive validity of the Foot Posture Index (FPI6) relative to the navicular drop test (NDT) was also assessed. **Methods:** Fifty-nine athletes participated: 10 in a reliability subsample and 49 in a separate validity subsample. Assessments included BMI, FPI-6, hallux valgus angle, hallux dorsiflexion, Y Balance Test (YBT), Q-angle, and NDT. Reliability was assessed using intraclass correlation coefficients (ICCs). Associations among measures and FPI6-NDT concurrent validity were examined using Spearman correlations and non-parametric group comparisons. Classification performance indices relative to NDT were calculated for FPI-6. **Results:** All measures demonstrated good to excellent reliability (ICC = 0.81–0.99). FPI-6 showed a strong concurrent association with NDT (rho = 0.735). More pronated foot posture was moderately associated with lower YBT performance and reduced hallux dorsiflexion (rho = −0.301 to −0.635). Higher BMI was associated with greater navicular drop and lower dynamic balance (rho = 0.301 to −0.433). FPI-6 showed high classification agreement with NDT-defined foot posture groups (specificity 96.9%; overall agreement 91.8%). **Conclusions:** Lower extremity alignment, hallux mobility, dynamic balance, and BMI showed modest interrelationships. FPI-6 demonstrated strong concurrent validity and high classification agreement relative to NDT, supporting practical screening use. Given the correlational nature of the findings, interpretations should remain cautious, and larger multimodal studies are warranted.

## 1. Introduction

Participation in competitive collegiate athletics has increased substantially in recent years, a trend that parallels report of rising sports-related lower-extremity injuries [1]. Lower-extremity malalignment has been identified as an important contributor to conditions such as patellofemoral pain, tendinopathies, anterior cruciate ligament injury, medial tibial stress syndrome, stress fractures, plantar fasciitis, and various forefoot disorders [2,3]. For this reason, numerous studies have examined abnormal anatomical alignments and their biomechanical interactions to better understand the potential pathways to injury or reinjury [3,4,5,6]. However, the literature also documents inconsistency across static lower extremity measurements—such as pelvic tilt, femoral anteversion, quadriceps angle, tibiofemoral angle, genu recurvatum, tibial torsion, and navicular drop—likely due to variations in examiner experience, difficulties with anatomical landmark identification, and instrument-related measurement errors [7]. Comprehensive static assessment of lower-extremity alignment is also time-intensive and often impractical in many clinical or athletic settings, particularly when multiple athletes require screening. Importantly, the aim of such screening approaches is not to predict injury in isolation but to identify modifiable risk patterns that can guide targeted preventive strategies. In this context, combining structural, functional, and anthropometric measures may provide greater clinical value than relying on any single metric alone. Given these limitations, there is substantial value in identifying assessment strategies built around modifiable intrinsic risk factors, including BMI, strength, flexibility, balance, and postural biomechanics [8,9,10,11]. Elevated body weight or BMI may increase injury risk yet can often be modified through targeted nutrition and physical training programs [12,13]. Many dynamic measures related to strength, balance, and neuromuscular control also respond well to structured training interventions [14]. Among available dynamic tests, Y Balance Test (YBT) provides a practical method of assessing dynamic balance in less than three minutes and has been shown to predict lower-extremity injury and identify athletes with chronic ankle instability [15,16].

Foot and hallux biomechanics also play an important role in lower-extremity function. Limited ankle dorsiflexion due to gastrocnemius tightness can increase tension through the Achilles–calcaneus–plantar system and alter first-metatarsophalangeal (MTP) joint mechanics, contributing to hallux limitus [17]. Restricted hallux motion can increase plantar pressure beneath the first metatarsal head [18] and interfere with athletic propulsion, possibly raising the risk of turf-toe and other forefoot injuries [19]. Tightness in plantar structures or compensatory patterns may further influence dorsiflexion and even contribute to hallux valgus [20]. Conservative management strategies—including orthoses and targeted muscle-balance exercises—have demonstrated reduction in hallux valgus angle in mild and moderate cases [21]. Because foot posture is modifiable and clinically relevant, several methods exist for foot-type classification, including footprint tests, radiographs, the navicular drop test (NDT), and the Foot Posture Index-6 (FPI-6) [22]. The FPI-6 visually evaluates multiple foot segments and soft-tissue contours, whereas the NDT measures changes in navicular height and is often recommended as a primary classification method owing to its structural focus [22,23,24]. Radiographic assessment, although precise, is rarely feasible for routine screening due to cost and radiation exposure concerns [25]. Orthotic intervention has also been shown to modify foot pronation and reduce the quadriceps angle in individuals with pronated feet [26,27], and a larger Q-angle itself has been associated with increased injury risk [28].

These measures are not conceptually independent. Foot posture influences the length–tension relationship of the plantar and Achilles–calcaneal structures, which in turn affects hallux dorsiflexion and forefoot loading during propulsion [17,18]. Altered foot posture and restricted hallux motion may also plausibly compromise the base of support and proprioceptive input required for dynamic balance tasks such as the YBT, a relationship consistent with the broader role of neuromuscular control in dynamic balance performance [15,16]. BMI can influence this chain further; as greater body mass is associated with reduced postural stability [12]. However, it remains unclear whether BMI is directly associated with navicular drop, as findings on this specific relationship have been inconsistent across studies [29]. The Q-angle, while primarily reflecting proximal alignment, has also been associated with compensatory distal foot mechanics [4,6]. Because these structural, functional, and anthropometric factors are mechanically and clinically interrelated, evaluating them in isolation may understate their combined contribution to injury risk; a single integrated assessment battery may therefore better reflect real-world screening needs than any single measure alone.

The relationship between structural alignment and functional performance may differ meaningfully between athletes and the general population. Repetitive high-volume loading, sport-specific movement demands, and years of neuromuscular adaptation can alter how static malalignments translate into dynamic function, potentially masking or amplifying their functional consequences [14]. Athletes may also develop compensatory motor strategies that reduce the practical impact of structural asymmetries observed in sedentary individuals, or conversely, repetitive loading patterns specific to certain sports may exacerbate the functional consequences of pre-existing malalignment. Because screening tools developed or validated primarily in general or clinical populations may not directly generalize to athletes, evaluating these measures and their interrelationships specifically within a collegiate athletic population is necessary to inform sport-relevant screening and injury-prevention strategies.

In multi-disciplinary environments, quick, practical, and reliable assessments that capture modifiable intrinsic factors together with dynamic assessments are especially valuable. Accordingly, this study aimed to: (1) establish intra- and inter-rater reliability of FPI6, hallux valgus angle, hallux dorsiflexion, Q-angle, YBT, and NDT in collegiate athletes; (2) examine relationships among BMI, hallux angles, foot type (via FPI6 and NDT), dynamic balance (YBT), and Q-angle; and (3) determine the concurrent, discriminant, and predictive validity of the FPI6 compared with the NDT in healthy college athletes.

## 2. Materials and Methods

### 2.1. Participants and Ethical Approval

**Figure 1:**
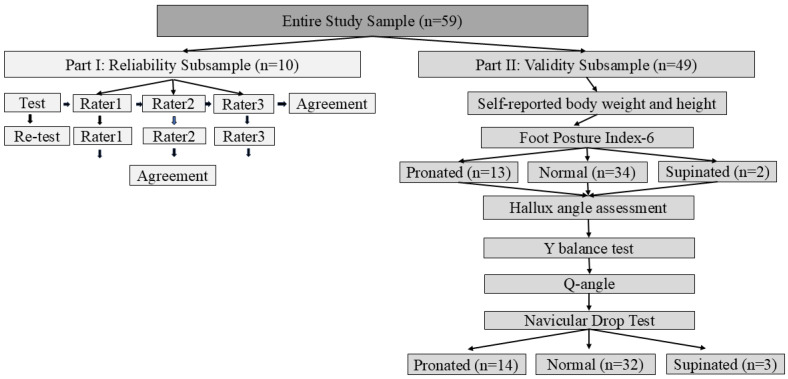
The research design and flow of participants throughout the study.

This cross-sectional study recruited 59 college athletes between October 2023 and May 2024 via team and club announcements and word-of-mouth. Athletes were divided into two non-overlapping subsamples: 10 athletes completed reliability testing only, and the remaining 49 athletes completed validity procedures only (10 + 49 = 59) (Figure 1). The reliability subsample size mirrored prior reliability work [23]. The validity subsample size was calculated with G*Power 3.1.9.7 for χ^2^ tests at α = 0.05 and 80% power, using the FPI6–NDT correlation reported in college students with low arches [22,30]. Inclusion criteria were age ≥18 years, university enrollment, and active participation in organized sport; exclusion criteria included medical restriction, injury limiting testing, recent lower-extremity surgery, or pain preventing participation. Ethical approval was obtained from Koç University, and all procedures followed the Declaration of Helsinki with written informed consent.

### 2.2. Study Variables and Procedure

All measurements were performed on the dominant leg to avoid inflated type-I error from pooling bilateral data [31]. BMI was calculated from self-reported height and weight. Foot posture was assessed first with the FPI-6, which visually grades six postural features; total scores classify feet as pronated (≥8), neutral (0–5), or supinated (≤−1) [32]. Prior studies indicate that even novice examiners can achieve acceptable reliability with FPI-6 after minimal training, and substantial-to-almost-perfect agreement has been reported for foot-type classification [33,34].

Hallux assessments were performed with a standard goniometer in weight-bearing. HVA was measured with the fulcrum at the first MTP joint and arms aligned to the first metatarsal and proximal phalanx; previous work supports intra-rater repeatability and reports moderate agreement with radiographic angles [35,36]. HLA—defined as the weight-bearing active maximum first-MTP dorsiflexion angle—was measured with one arm aligned to the first metatarsal and the other to the proximal phalanx; reported reliability is moderate-to-good overall and improves to excellent with experienced examiners [37,38]. Each measure was recorded twice and averaged.

Dynamic balance was assessed with the Y Balance Test following standard practice and scoring. Participants completed three trials in the anterior, posteromedial, and posterolateral directions; the best distances were used to calculate a composite score normalized to limb length. Reported interrater test–retest reliability for maximal reach is good among raters with limited experience, and composite reach scores demonstrate high intra-/inter-rater reliability in collegiate athletes [39,40].

The Q-angle was measured in relaxed stance using ASIS, patellar midpoint, and tibial tuberosity as landmarks; the measured angle has been linked to injury risk in some populations, and standardized goniometric protocols yield good intra-rater reliability [28,41]. Foot posture was also quantified structurally using the NDT, calculated as the change in navicular height between subtalar-neutral non-weight-bearing and full weight-bearing; thresholds used were >9 mm (pronated), 5–9 mm (neutral), and <5 mm (supinated), with excellent reported intra- and inter-rater reliability [22].

Three medical student examiners completed five structured training sessions covering palpation, measurement technique, and protocol standardization under supervision of a medical doctor. Reliability testing was conducted in two same-day sessions with ≥10 min rests; foot-placement templates standardized stance across sessions. Raters were blinded to one another’s and to their own prior readings. For the validity phase, the fixed order was BMI, FPI-6, HVA, HLA, YBT, Q-angle, and NDT.

### 2.3. Statistical Analysis

Analyses were performed in SPSS v29. ICCs (two-way mixed, absolute-agreement) quantified intra-/inter-rater reliability and were interpreted using conventional thresholds (poor ≤0.50, moderate 0.50–0.75, good 0.75–0.90, excellent ≥0.90) [42]. Data normality was evaluated with Shapiro–Wilk. Spearman coefficients summarized associations and concurrent validity (FPI-6 vs. NDT), with correlation strength interpreted as strong >0.70, moderate 0.30–0.70, weak <0.30 [43]. Kruskal–Wallis tested discriminant validity of FPI-6 across NDT-defined groups with Mann–Whitney U for pairwise comparisons. Sensitivity, specificity, likelihood ratios, and diagnostic accuracy summarized predictive validity (normal vs. abnormal). Ordinal regression estimated variance in NDT explained by FPI-6.

## 3. Results

### 3.1. Rater Reliability

Ten athletes (five male and five female) participated in the reliability phase. Each participant was evaluated twice by three examiners in sessions lasting 90–120 min, with approximately one hour of rest between the test and retest. The intra-rater reliability values for all continuously scored measures ranged from 0.813 to 0.990, indicating good to excellent reliability. Inter-rater reliability values were similarly strong, ranging from 0.819 to 0.986 (Table 1). These results demonstrated that the three trained raters produced highly consistent measurements across sessions and examiners.

### 3.2. Concurrent Validity and Correlations

Forty-nine athletes completed all measurements in the validity session of the study. The median age was 21 years (range 18–25), and most participants were male (67.3%). Athletes represented multiple sports, including American football, frisbee, rugby, basketball, and taekwondo, with an average of two years of active collegiate competition.

All correlations between measurements were shown in Table 2. A strong positive correlation was found between FPI-6 and NDT scores (rho = 0.735, *p* < 0.01), demonstrating strong concurrent validity between the two foot-type classification methods. Moderate negative correlations were observed between FPI-6 and YBT scores (rho = −0.327, *p* < 0.05) and between NDT and YBT scores (rho = −0.635, *p* < 0.01), indicating that more pronated foot postures were associated with lower dynamic balance performance. Pronated foot types were also associated with reduced hallux dorsiflexion, reflected in the moderate negative correlations between FPI-6 and HLA (rho = −0.301, *p* < 0.05) and between NDT and HLA (rho = −0.415, *p* < 0.01).

BMI showed a moderate positive correlation with NDT (rho = 0.301, *p* < 0.05), suggesting that higher BMI may be associated with greater navicular drop and therefore lower arch height. A moderate negative correlation was also found between BMI and YBT (rho = −0.433, *p* < 0.01), indicating that increased BMI negatively influenced dynamic balance. Additionally, a moderate positive correlation was observed between HLA and YBT performance (rho = 0.336, *p* < 0.05).

### 3.3. Discriminant Validity

FPI-6 scores differed significantly among the pronated, neutral, and supinated foot-type groups classified by NDT (H(2) = 29.361, *p* < 0.001). Pairwise comparisons showed that participants with pronated feet had significantly higher FPI-6 scores than those with neutral feet, and neutral-footed participants had significantly higher FPI-6 scores than those with supinated feet. The same pattern was observed between the pronated and supinated groups. Median FPI-6 scores were 7 for the pronated group, 2 for the neutral group, and –1 for the supinated group. These results confirmed that the FPI-6 effectively distinguished between foot-type categories defined by the NDT.

### 3.4. Predictive Validity

**Figure 2:**
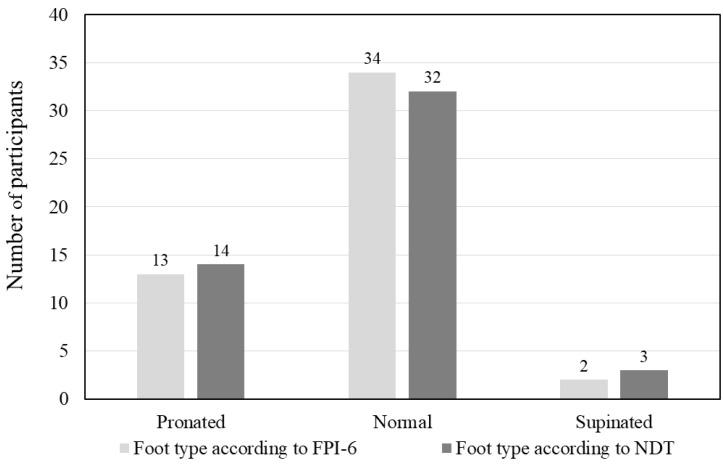
Foot type classification according to FPI-6 and NDT.

Based on the NDT, 32 athletes were classified as having a normal foot type and 17 as having an abnormal foot type (pronated or supinated). The FPI-6 correctly identified 31 of the 32 athletes with normal foot type and 14 of the 17 athletes with abnormal foot type (Figure 2). Accordingly, the FPI-6 demonstrated a sensitivity of 82.4% (95% CI: 58.97–93.81%), a specificity of 96.9% (95% CI: 84.26–99.45%), and an overall classification accuracy of 91.84% for identifying normal versus abnormal foot types. The FPI-6 score alone explained 83.36% of the variance in NDT classifications (Nagelkerke R^2^). These results highlighted its strong potential as a practical tool for foot-type screening in athletic populations.

## 4. Discussion

This study yielded two main findings. First, the FPI6 demonstrated strong concurrent validity and high classification agreement relative to the NDT, supporting its use as a practical, reliable tool for foot-type screening in collegiate athletes. Second, foot posture, hallux mobility, dynamic balance, and BMI showed meaningful interrelationships, indicating that these modifiable intrinsic factors are not independent but instead reflect a connected pattern relevant to athletic screening. All measures also demonstrated good-to-excellent reliability (ICC = 0.81–0.99), supporting the trustworthiness of the set of measures as a whole.

This pattern aligns with prior reports showing that structured examiner training and clear protocols can yield dependable performance on the FPI-6, hallux goniometry, Y-Balance, and Q-angle assessments [33,34,38,39,40,41]. Slightly lower agreement for the NDT and Q-angle in our setting may reflect practical challenges such as precise landmark palpation and maintaining consistent lower-limb alignment, which have also been discussed previously [22,41].

A central finding of this study was the strong concurrent validity between FPI-6 and NDT scores. Although the two tools differ in emphasis—FPI-6 captures multi-segment visual features of rearfoot, midfoot, and forefoot, whereas the NDT quantifies arch-height change—our findings suggest that they reflect related but not identical aspects of foot posture in this population. Similar convergence between visual/postural indices and midfoot height measures has been described previously, supporting the view that simple clinical tools can provide a reasonable proxy for structural alignment in screening contexts [22,31]. This convergence extended to classification: FPI6 scores differed significantly across NDT-defined pronated, neutral, and supinated groups, and FPI6 showed high specificity and overall classification agreement for identifying normal foot type, though sensitivity was more modest. Together, these findings indicate that FPI6 can capture category-level differences in foot posture in a manner broadly consistent with the NDT, making it an attractive point-of-care option in time-limited screening scenarios [44]. Still, it would be premature to treat the tools as interchangeable in all settings; observational grading may be more susceptible to examiner experience, whereas the NDT can be influenced by soft-tissue characteristics and the accuracy of subtalar neutral positioning [45], and the more modest sensitivity of FPI-6 suggests that some abnormal postures may be missed if used alone. Where a more comprehensive evaluation is necessary, combining FPI-6 with an objective measure such as the NDT—or, when feasible, radiographic benchmarks—may reduce classification error [25]. Overall, these accessible tools need not be exclusive choices; they can be layered strategically to enhance clinical confidence, particularly in resource- or time-constrained settings.

Relationships between foot posture, hallux dorsiflexion, and dynamic balance were also noteworthy. In our sample, more pronated foot posture tended to accompany lower Y-Balance performance and reduced first MTP dorsiflexion. While the cross-sectional design limits causal inference, these patterns are best interpreted as associations rather than indicators of direct mechanical influence, though they align with biomechanical considerations: increased tension within the Achilles–calcaneus–plantar system and altered forefoot mechanics in pronation may constrain hallux motion and challenge postural control during weight-bearing tasks [17,18]. Given the role of the hallux in propulsion and stability, even small restrictions in dorsiflexion may have functional consequences in athletic movements that rely on repeated push-off and rapid direction changes, making hallux mobility a practical and low-burden addition to on-field screening protocols [19]. Contrary to our findings, Moreno-Barriga et al. (2023) reported no significant differences in static or dynamic postural stability across athletes with low-, normal-, or high-arched feet, suggesting that foot structure may not meaningfully influence balance in trained athletic populations [46]. The authors proposed that neuromuscular compensatory mechanisms developed through regular sports participation may mask the potential destabilizing effects of altered foot posture. In contrast, the moderate negative associations observed in our study between pronated foot posture (higher FPI-6 and NDT values) and Y-Balance Test performance indicate that altered foot alignment can meaningfully affect dynamic stability. These divergent results may be explained by methodological differences: whereas the current study used posture-oriented measures (FPI-6 and NDT) and a functional dynamic balance assessment, Moreno-Barriga et al. relied on a static footprint-based arch index and posturographic sway parameters, which may be less sensitive to functional deficits. Taken together, these findings highlight that the influence of foot posture on balance may vary depending on the sensitivity of the measurement tools and the extent of neuromuscular adaptation in athletic populations. Consistent with our findings, Žnidaršič et al. (2024) showed that a lower medial arch (higher arch index) was associated with poorer balance performance, including longer time-to-stabilization and increased postural corrections during single-leg tasks, supporting the notion that pronated foot posture may compromise dynamic balance [47]. Together, these observations suggest that clinicians may benefit from prioritizing quick screens that integrate foot posture with functional tests when evaluating at-risk athletes.

Findings involving BMI warrant a nuanced interpretation. Higher BMI showed moderate associations with greater navicular drop and lower Y-Balance performance in our sample, although BMI was not related to FPI-6. Because BMI does not distinguish lean from fat mass—an especially relevant caveat in an athletic population, where higher BMI may reflect greater muscle mass rather than adiposity—these relationships should be interpreted cautiously. One possibility is that soft-tissue distribution influences measured navicular height more readily than visually rated posture features, contributing to the discrepancy between NDT and FPI-6 with respect to BMI; in individuals with higher BMI, relying solely on visual evaluation to classify foot type may therefore lead to misclassification, whereas an objective measurement such as the navicular drop test may provide a more reliable assessment in these cases. Independent studies linking balance performance and BMI to ankle sprain risk further underscore the value of monitoring dynamic balance alongside anthropometrics in collegiate populations [48], suggesting that screening approaches may benefit from combining a structural or quasi-structural marker with a functional test rather than relying on a single index.

Methodological points also deserve attention. Reliability for hallux dorsiflexion is known to vary with examiner experience and technique; while our training procedures likely contributed to consistent intra- and inter-rater estimates, clinicians should remain mindful that joint-specific positioning and patient effort can introduce variability [37,38]. Similarly, small differences in Q-angle technique (e.g., foot position, quadriceps relaxation, patellar centering) can influence readings; maintaining standardized positioning, as implemented here, is advisable to mitigate measurement noise [41]. For dynamic balance, the observed associations with foot posture and BMI are clinically plausible, but replication with sport-specific balance and agility tasks could clarify how general balance screens translate to on-field risk in different sports [39,40]. Finally, given that orthotic interventions can influence knee alignment and address foot posture, future work might explore whether targeted orthoses meaningfully alter hallux dorsiflexion or dynamic balance in athletes who screen positive for pronation [26,27].

### 4.1. Practical Applications

From a clinical perspective, the present results support the use of simple, accessible tools to build an integrated view of intrinsic risk factors. In contexts such as pre-participation exams or in-season monitoring, pairing a quick visual classification (FPI-6) with a functional measure (Y-Balance) and, where indicated, a structural index (NDT) may provide a more complete screen than any single test alone. Considering the potential influence of BMI on dynamic balance and navicular height, incorporating basic anthropometrics and weight-management counseling where appropriate may also be useful. Importantly, clinicians might prioritize patterns across measures rather than single thresholds—for instance, a combination of pronated posture, reduced hallux dorsiflexion, and lower composite Y-Balance performance indicates a high injury risk and may justify early corrective strategies such as mobility work, intrinsic foot strengthening, and orthotic support [17,32,47].

### 4.2. Limitations

This study has several limitations that should temper interpretation. BMI was calculated from self-reported height and weight. As BMI does not distinguish fat mass from muscle mass, and self-reported anthropometric data may be subject to reporting bias, BMI values in this athletic cohort should be interpreted as an approximate rather than precise measure of body composition. The sample consisted of volunteers from a single collegiate environment and may not generalize to other levels of competition or to non-athletes. Assessments were limited to the dominant foot; potential bilateral asymmetries were therefore not captured. Radiographic imaging was not included, so comparisons with structural “gold-standard” measures were not possible; prior work suggests that clinical tests can approximate posture categories, but imaging can still be informative where differential diagnosis or surgical planning is concerned [24]. The number of supinated feet was relatively small, potentially limiting power for between-group contrasts. Additionally, reliability analyses were based on a relatively small subsample, which may affect the precision of ICC estimates. While the sample size was comparable to previous reliability studies, the findings should be interpreted cautiously. Lastly, although standardized rest periods and test sequences were used, conducting all validity measurements within a single session leaves some potential for fatigue, motivation, or short-term learning effects. These design features are common in pragmatic screening studies but should be acknowledged in translating findings to practice.

## 5. Conclusions

In summary, this study demonstrated that lower-extremity screening measures—including FPI6, HVA, HLA, YBT, Q-angle, and NDT—can be applied reliably in collegiate athletes and that FPI6 shows strong concurrent validity, discriminant ability, and classification agreement relative to NDT, supporting its practical use as a foot-type screening tool. Foot posture, hallux mobility, dynamic balance, and BMI showed meaningful interrelationships, indicating that these modifiable intrinsic factors are best evaluated together rather than in isolation. Taken together, these findings support combining visual, structural, and functional assessments into a multimodal screening approach for collegiate athletes, rather than relying on any single measure. Larger, more diverse, and longitudinal studies incorporating bilateral assessments and structural imaging would help clarify underlying mechanisms and refine practical guidance for athlete screening and injury-prevention strategies [22,24,25].

## Figures and Tables

**Table 1:** Intra and inter-rater reliability.

Measurements	Intra-Rater Reliabilities	Inter-Rater Reliability
	ICC Range	ICC
FPI-6 score	0.915–0.990	0.986
HLA	0.943–0.968	0.954
HVA	0.890–0.961	0.906
YBT	0.935–0.961	0.974
Q-angle	0.813–0.893	0.819
NDT score	0.863–0.911	0.890

ICC: Intraclass correlation coefficient; FPI-6: Foot posture index; HLA: Hallux limitus angle; HVA: Hallux valgus angle; YBT: Y-Balance test; NDT: Navicular drop test.

**Table 2:** Correlation coefficient matrix among measurements.

Measurements	FPI-6 Score	HLA	HVA	YBT	Q-Angle	NDT
**BMI**	0.206	0.005	−0.006	−0.433 **	0.234	0.301 *
**FPI-6 score**		0.085	−0.301 *	−0.327 *	−0.056	0.735 **
**HVA**			0.219	−0.079	0.069	0.141
**HLA**				0.336 *	−0.072	−0.415 **
**YBT**					−0.027	−0.635 **
**Q-angle**						−0.042

* *p* < 0.05: significant correlation coefficients, ** *p* < 0.01: significant correlation coefficients.

## Data Availability

The data presented in this study are not publicly available due to privacy and ethical restrictions. The dataset includes individual-level demographic and anthropometric information (age, gender, sport type, and body measurements) collected from a relatively small, identifiable cohort of collegiate athletes at a single institution. Given the small sample size and the specificity of the population, there is a meaningful risk that individual participants could be re-identified from the combination of variables, even without direct identifiers such as names. In addition, participant consent and institutional ethical approval covered the specific aims of this study and did not include provisions for public deposition of individual-level data. Data are available from the corresponding author upon rea-sonable request, subject to a data-sharing agreement and any necessary institutional/ethical approval.

## Abbreviations

The following abbreviations are used in this manuscript:

BMI	Body Mass Index
FPI-6	Foot Posture Index-6
NDT	Navicular Drop Test
HLA	Hallux Limitus Angle
HVA	Hallux Valgus Angle
YBT	Y-Balance Test
ICC	Intraclass coefficient
MTP	Metatarsophalangeal
